# Fungi as a Potential Source of Pigments: Harnessing Filamentous Fungi

**DOI:** 10.3389/fchem.2020.00369

**Published:** 2020-05-08

**Authors:** Rishu Kalra, Xavier A. Conlan, Mayurika Goel

**Affiliations:** ^1^Division of Sustainable Agriculture, TERI-Deakin Nanobiotechnology Centre, The Energy and Resources Institute, Gurugram, India; ^2^School of Life and Environmental Sciences, Deakin University, Geelong, VIC, Australia

**Keywords:** fungi, pigments, endophytes, lichen, halophytes

## Abstract

The growing concern over the harmful effects of synthetic colorants on both the consumer and the environment has raised a strong interest in natural coloring alternatives. As a result the worldwide demand for colorants of natural origin is rapidly increasing in the food, cosmetic and textile sectors. Natural colorants have the capacity to be used for a variety of industrial applications, for instance, as dyes for textile and non-textile substrates such as leather, paper, within paints and coatings, in cosmetics, and in food additives. Currently, pigments and colorants produced through plants and microbes are the primary source exploited by modern industries. Among the other non-conventional sources, filamentous fungi particularly ascomycetous and basidiomycetous fungi (mushrooms), and lichens (symbiotic association of a fungus with a green alga or cyanobacterium) are known to produce an extraordinary range of colors including several chemical classes of pigments such as melanins, azaphilones, flavins, phenazines, and quinines. This review seeks to emphasize the opportunity afforded by pigments naturally found in fungi as a viable green alternative to current sources. This review presents a comprehensive discussion on the capacity of fungal resources such as endophytes, halophytes, and fungi obtained from a range or sources such as soil, sediments, mangroves, and marine environments. A key driver of the interest in fungi as a source of pigments stems from environmental factors and discussion here will extend on the advancement of greener extraction techniques used for the extraction of intracellular and extracellular pigments. The search for compounds of interest requires a multidisciplinary approach and techniques such as metabolomics, metabolic engineering and biotechnological approaches that have potential to deal with various challenges faced by pigment industry.

## Introduction

Color is both the first and most pleasing feature an individual notices when approaching food and this feature is known to be associated with the flavor, safety, and nutritional value of the food item in question (Pathare et al., 2013; Spence, 2015). This has been a consistent theme throughout history with the ancient Egyptians and Romans around 1500 B.C describing the concept food colors and how they relate to bioactivity (Adam Burrows, 2009). Initially nature was the only source of food colors (Rohrig, 2016; Yusuf et al., 2017), however, the high cost of extraction and instability of the traditionally natural colors drove the development of synthetic colors during the 1800s. Perkin's mauve pigment was the first synthetic color discovered by English chemist Sir William Henry Perkin in 1856 (Garfield, 2002). This discovery led to the foundation of new era of synthetic dyes commonly known as “coal-tar colors,” derived from aniline and other organic compounds. These synthetic colors were widely used in industries having an impact in textiles, cosmetics and pharmaceuticals (Morris and Travis, 1992; Adam Burrows, 2009). More, serious apprehensions against synthetic colors were raised in 2007 after a study at University of Southampton highlighted the link between some artificial colors and hyperactivity in children (McCann et al., 2007; Arnold et al., 2012). These colors known as “the Southampton six” including sunset yellow FCF (E110), quinolone yellow (E104), carmoisine (E122), allura red (E129), tartrazine (E102), and ponceau 4R (E124) became the focus of the impact of colorants on humans.

In 2010, European Union (EU) regulators directed compulsory warnings on children's food labeling and recommended the limited acceptable daily consumption levels of some colourings. Similarly, organizations like the United States Food and Drug Administration (US-FDA) and World Health Organization (WHO) also regulated the usage of these colors in food, drugs, and cosmetic items. With the advent of the various issues associated with the over use of synthetic pigments, intense research in natural color dyes has been initiated in recent years (Mapari et al., 2010; Aberoumand, 2011; FDA, 2011; Harasym and Bogacz-Radomska, 2016; Mehrad et al., 2018; Duarte et al., 2019).

Natural food coloring is a major focus of the modern food manufacture industry and is an ever growing market allowing an increase in research in this space (Faustino et al., 2019). This has been led by growing customer awareness and demand for products without synthetic colorants and has fueled the growth of natural color industry. Natural food color not only gives an appealing and appetizing look but might also possesses nutritional and health benefits (Delgado-Vargas and Paredes-Lopez, 2003; Bora et al., 2019). Nature has always been considered a treasure trove of organisms comprising plants, animals, and microorganisms with a capacity to produce pigments. Some of the established natural pigments that are frequently employed to provide color to food and are considered safe include anthocyanins, carotenoids, betalains, chlorophylls, and curcumin (Mortensen, 2006; Socaciu, 2007; Gengatharan et al., 2015; Janiszewska-Turak et al., 2016; Corrêa et al., 2019). These components not only provide the coloring property to the food industry but also enhance the nutritious and pharmacological potential of the food product through primarily acting as antioxidants. Detailed studies on the characteristics and properties of these natural pigments has been conducted and discussed by research groups, however the potential of these pigments as a source of new commercial pigments is limited by a number of factors. Some of the challenges which the natural color industry is facing include raw material availability and the stability and sensitivity of these natural pigments toward various external parameters (Scotter, 1995; Mercadante, 2007; Galaffu et al., 2015). To better understand the opportunity for colors from natural sources what is known about the well-studied plants and animal sources needs to be extended into microorganisms which have the potential as a source for biopigments production (Sen et al., 2019).

Pigments produced from microbial origin have several advantages over those obtained from plant or animal including supply sustainability; yield; cost efficiency; stability; labor cost and ease of downstream processing (Joshi et al., 2003; Tuli et al., 2015). Innumerable reports are available on the application of various biotechnological tools for the isolation of a plethora of new colors from microbial origin (Joshi and Attri, 2005; Rymbai et al., 2011; Gharibzahedi et al., 2012). Among various microbial alternatives, microalgae and fungi produce a remarkable range of water soluble biopigments that have a range of ecological functions (Gmoser et al., 2017; Heer and Sharma, 2017), however, lower harvest yields of the algal cultures is the major bottleneck to exploit its potential for commercial production (Hejazi and Wijffels, 2004). In efforts to utilize fungi for biopigment production, basidiomycetous fungi which have been utilized by ancient cultures for dying silk and wool (Hernández et al., 2019) have been studied. However, bulk production of these fungi for commercial purpose is not feasible. Thus, the industry is more focussed on filamentous fungi which can be easily grown in lab and allowed for the opportunity for large scale production. This include fungi from a broad range of environments of marine origin, soil endophytic fungi from terrestrial and marine flora and endolichenic fungus (Mapari et al., 2010; Gao et al., 2013; Dufosse et al., 2014).

Many colors produced by the filamentous fungi for example ankaflavin and canthaxanthin from *Monascus* sp. and Arpink red™ pigment (Natural red™) from the strain *Penicillium oxalicum var. armeniaca* are already in the market (Mapari et al., 2009). Though natural colors have taken a lead in comparison to the synthetic ones in a rapidly changing industry, the exploration of fungal based pigments still needs attention toward their potential as future industrial pigment. This review will highlight the potential filamentous fungi sources that have the capacity to be explored in order to produce these pigments, their application as commercial natural colorants and challenges faced by the industry for the commercial application of these pigments. Focus will be given to the advanced analytical techniques that are currently used to identify novel pigment components and how these techniques can be applied to study the biosynthetic pathway. In addition to these, metabolic engineering approaches that have the potential to enable the mass production of the fungal resources of colorants for a broad range of industries will also be studied. Further newer greener and sustainable extraction techniques used for the isolation of the pigments for industrial application will be explored highlighting the emerging green economy.

## Fungi as a Source of Pigments

Increasing demand, limited resources and various disadvantages associated with the currently authorized natural pigments such as limited availability throughout the year, instability against light, heat and adverse pH, variation in pigment extraction and low water solubility, demands new research into the sustainable sources of natural colorants. Recently, fungi have attracted special attention for the production of natural pigments based on the fact that they contain compounds with high light and chemical stability, a spectrum of colors, high yield and a sustainable supply (Durán et al., 2002; Fouillaud et al., 2016; Chen et al., 2017).

**Figure 1:**
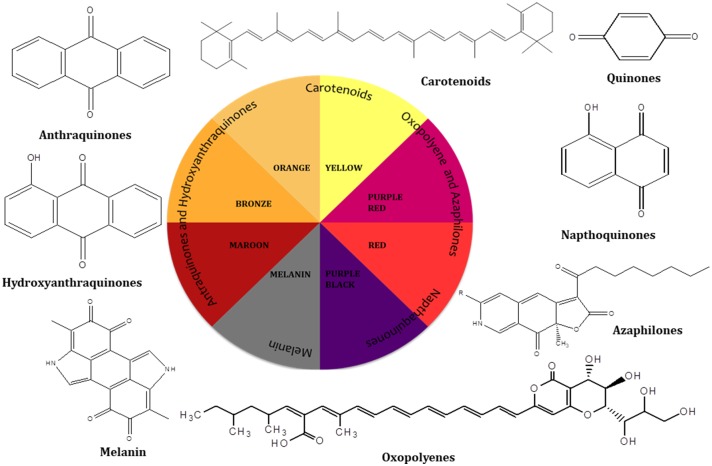
Different hues of colors produced by different classes of compounds.

Pigments production by fungal colonies has kindled interest among mycologist since the 19th century and can be considered a microbial reserve for the production of food grade pigments. Fungi are known to produce a wide array of pigments which includes metabolites from several classes such as melanins, anthraquinones, hydroxyanthraquinones, azaphilones, carotenoids, oxopolyene, quinones and naphthoquinone (Figure 1) (Mapari et al., 2005; Osmanova et al., 2010; Xie et al., 2016; Chuyen and Eun, 2017; Pombeiro-Sponchiado et al., 2017). In Figure 1 some of the shades and colors hues produced by different class of metabolites and their basic chemical structures can be observed. Arpink red™ pigment (Natural red™) which is the first commercial red color from a fungus has been produced from the strain *Penicillium oxalicum var. armeniaca* CCM 8242 isolated from soil (Caro et al., 2017). Biosynthetically, many of these pigments are polyketides derivatives which are produced abundantly in the majority of the ascomycetous fungi. g *Neurospora* spp. and *Monascus* spp. are such examples of ascomycetes fungi highlighting a key area for further development. Besides, polyketide based molecules; other classes of pigmented metabolites present in various filamentous fungi are terpenoids, polyphenols, and carotenoids. To gain an understanding of the breadth of color afforded by these molecules some of the common pigments produced by these species are displayed in Table 1.

**Table 1:** Examples of selected pigments produced by different fungal species grouped with respect to genus producing them.

**S.no**.	**Fungal/lichen species**	**Pigment**	**Class of compound**	**Pigments color**	**Molecular formula and weight**	**References**
1.	*Alternaria solani, Alternaria porri, Alternaria tomatophila*	Altersolanol A	Hydroxyanthraquinone	Yellow	C_16_H_16_O_8_; 336.3	Andersen et al., 2008
2.	*Alternaria sp. ZJ9-6B*	Alterporriol K	Anthraquinone	Red	C_32_H_26_O_10_; 586.14	Huang et al., 2011
3.	*Alternaria sp. ZJ9-6B*	Alterporriol L	Anthraquinone	Red	C_32_H_26_O_12_; 602.54	Huang et al., 2011
4.	*Alternaria sp. ZJ9-6B*	Alterporriol M	Anthraquinone	Red	C_32_H_25_O_12_; 601.13	Huang et al., 2011
5.	*Alternaria sp. ZJ9-6B*	Macrosporin	Hydroxyanthraquinone	Yellow	C_16_H_12_O_5_; 284.2	Huang et al., 2011
6.	*Alternaria sp. ZJ9-6B*	Dactylariol	Hydroxyanthraquinone	Not mentioned	C_16_H_16_O_7_; 320.2	Huang et al., 2011
7.	*Alternaria sp. ZJ9-6B*	Tetrahydroaltersolanol B	Hydroxyanthraquinone	Not mentioned	C_16_H_20_O_6_; 308.3	Huang et al., 2011
8.	*Alternaria sp. (SK11)*	Alterporriols C	Hydroxyanthraquinone	Orange	C_32_H_26_O_13;_ 618.5	Xia et al., 2014
9.	*Amygdalaria panaeola*	Panaefluorolines A	Isoquinoline	Yellowish green	C_19_H_24_${\text{NO}}_{4}^{+}$; 330.4	Kinoshita et al., 2003
10.	*Amygdalaria panaeola*	Panaefluorolines B	Isoquinoline	Yellowish green	C_17_H_20_${\text{NO}}_{4}^{+}$; 302.34	Kinoshita et al., 2003
11.	*Amygdalaria panaeola*	Panaefluorolines C	Isoquinoline	Yellowish green	C_18_H_22_${\text{NO}}_{4}^{+}$; 316.4	Kinoshita et al., 2003
12.	*Aspergillus sulphureus*	Viopurpurin	Naphtoquinones	Purple	C_29_H_20_O_11_; 544.5	Durley et al., 1975
13.	*Aspergillus sulphureus*	Rubrosulfin	Naphtoquinones	Red	C_29_H_20_O_10_; 528.5	Durley et al., 1975; Stack et al., 1977
14.	*Aspergillus ochraceus*	Viomellein	Quinone	Reddish-brown	C_30_H_24_O_11_; 560.50	Stack and Mislivec, 1978
15.	*Aspergillus ochraceus*	Xanthomegnin	Dihydroisocoumarin	Orange	C_30_H_22_O_12_; 574.48	Stack and Mislivec, 1978; Frisvad et al., 2004,
16.	*Aspergillus glaucus*	Catenarin	Hydroxyanthraquinone	Red	C_15_H_10_O_6_; 286.2	Anke et al., 1980
17.	*Aspergillus glaucus*	Rubrocristin	Hydroxyanthraquinone	Red	C_16_H_12_O_6_; 300.3	Anke et al., 1980
18.	*Aspergillus glaucus, Aspergillus cristatus, Aspergillus repens*	Erythroglaucin	Hydroxyanthraquinone	Red	C_16_H_12_O_6_; 300.26	Durán et al., 2002
19.	*Aspergillus glaucus*	Aspergiolide B	Hydroxyanthraquinone	Red	C_26_H_17_O_9_ 473.08	Du et al., 2008
20.	*Aspergillus variecolor*	Variecolorquinone A	Quinone	Yellow	C_20_H_17_O_9_; 401.08	Wang et al., 2007
21.	*Aspergillus variecolor*	Variecolorquinone A	Quinone	Yellow	C_17_H_15_O_6_; 315.08	Wang et al., 2007
22.	*Aspergillus sp. strain 05F16*	Bostrycin	Anthraquinone	Red	C_16_H_16_O_8_; 336.29	Xu et al., 2008
23.	*Aspergillus sp. strain 05F16*	Tetrahydrobostrycin	Anthraquinone	Yellow	C_16_H_21_O_8_; 341.12	Xu et al., 2008
24.	*Aspergillus sp. strain 05F16*	1-deoxytetrahydrobostrycin	Anthraquinone	Yellow	C_16_H_21_O_7_; 325.12	Xu et al., 2008
25.	*Aspergillus fumigatus*	Melanin	1,8-dihydroxynaphthalene	Dark-brown	C_18_H_10_N_2_O_4_; 318.28	Gonçalves et al., 2012
26.	*Aspergillus niger*	Azanigerones B	Azaphilones	Yellow	C_21_H_28_O_6_; 376.44	Zabala et al., 2012
27.	*Aspergillus niger*	Azanigerones C	Azaphilones	Yellow	C_21_H_26_O_7_; 390.43	Zabala et al., 2012
28.	*Aspergillus glaucus*	Physcion	Hydroxyanthraquinone	Yellow	C_16_H_12_O_5_; 284.3	Gessler et al., 2013
29.	*Aspergillus nidulans*	Emodin	Hydroxyanthraquinone	Yellow	C_15_H_10_O_5_; 270.2	Gessler et al., 2013
30.	*Blakeslea trispora*	Lycopene	Carotenoids	Red	C_40_H_56_; 536.87	Wang et al., 2012
31.	*Blakeslea trispora*	β-carotene	Carotenoids	Yellow-orange	C_40_H_56_; 536.87	Yan et al., 2013
32.	*Curvularia lunata*	Chrysophanol	Hydroxyanthraquinone	Orange-red	C_15_H_10_O_4_; 254.2	Durán et al., 2002
33.	*Curvularia lunata*	Cynodontin	Hydroxyanthraquinone	Bronze	C15H10O6; 286.2	Durán et al., 2002
34.	*Curvularia lunata*	Erythroglaucin	Hydroxyanthraquinon	Red	C_16_H_12_O_6_; 300.26	Caro et al., 2017
35.	*Dreschlera teres, Dreschlera dictyoides, Dreschlera avenae*.	Helminthosporin	Hydroxyanthraquinone	Maroon	C_15_H_10_O_5_; 270.2	Durán et al., 2002
36.	*Dreschlera teres, Dreschlera dictyoides, Dreschlera avenae*.	Catenarin	Hydroxyanthraquinon	Red	C_15_H_10_O_6_; 286.2	Durán et al., 2002
37.	*Dreschlera teres, Dreschlera dictyoides, Dreschlera avenae*.	Tritisporin	Hydroxyanthraquinon	Brownish-red	C_15_H_10_O_4_; 254.2	Durán et al., 2002
38.	*Daldinia concentrica*	Daldinin A	Azaphilones	Yellow	C_15_H_18_O_5_Na; 301.1	Shao et al., 2008
39.	*Daldinia concentrica*	Daldinin B	Azaphilones	Yellow	C_15_H_18_O_4_; 285.1	Shao et al., 2008
40.	*Daldinia concentrica*	Daldinin C	Azaphilones	Yellow	C_15_H_21_O_4_; 265.1	Shao et al., 2008
41.	*Emericella purpurea*	Epurpurins A	Dicyanide derivatives	Greenish-yellow	C_28_H_28_N_2_O_2_; 424	Takahashi et al., 1996
42.	*Emericella purpurea*	Epurpurins B	Dicyanide derivatives	Greenish-yellow	C_23_H_20_N_2_O_2_; 356.19	Takahashi et al., 1996
43.	*Emericella purpurea*	Epurpurins C	Dicyanide derivatives	Greenish-yellow	C_18_H_12_N_2_O_2_; 288.08	Takahashi et al., 1996
44.	*Emericella falconensis and Emericella fructiculosa*	Falconensin A	Azaphilones	Yellow	C_23_H_23_O_6_Cl_2_; 466.33	Ogasawara et al., 1997
45.	*Emericella falconensis and Emericella fructiculosa*	Falconensin E	Azaphilones	Yellow	C_23_H_25_O_7_Cl; 448.9	Ogasawara et al., 1997
46.	*Eurotium amstelodami*	Erythroglaucin	Hydroxyanthraquinone	Red	C_16_H_12_O_6_; 300.26	Podojil et al., 1978
47.	*Eurotium repens*	Auroglaucin	Hydroquinones	Orange-red	C_19_H_22_O_3_; 304.4	Podojil et al., 1978
48.	*Eurotium repens*	Physcion	Hydroxyanthraquinone	Yellow	C_16_H_12_O_5_; 284.3	Podojil et al., 1978
49.	*Eurotium rubrum and Eurotium cristatum*	Rubrocristin	Hydroxyanthraquinone	Red	C_16_H_12_O_6_; 300.3	Anke et al., 1980
50.	*Eurotium chevalieri*	Flavoglaucin	Hydroquinones	Yellow	C_19_H_28_O_3_; 304.4	Ishikawa et al., 1984
51.	*Eurotium rubrum and Eurotium cristatum*	Catenarin	Hydroxyanthraquinone	Red	C_15_H_10_O_6_; 286.2	Durán et al., 2002
52.	*Eurotium sp*	Isodihydroauroglaucin	Hydroquinones	Orange	C_19_H_24_O_3_; 300.4	Gawas et al., 2002
53.	*Eurotium sp*.	Tetrahydroauroglaucin	Hydroquinones	Yellow	C_19_H_26_O_3_; 302.4	Gawas et al., 2002
54.	*Eurotium rubrum*	3-O-(α-D-ribofuranosyl)-questin	Hydroxyanthraquinone	Orange	C_21_H_19_O_9_; 415.10	Li et al., 2009
55.	*Eurotium rubrum*	Asperflavin	Hydroxyanthraquinone	Greenish	C_16_H_16_O_5_; 288.29	Li et al., 2009
56.	*Eurotium rubrum*	2-O-methyleurotinone	Eurotinone analogs	Not mentioned	C_16_H_14_O_6_; 302.27	Li et al., 2009
57.	*Fusarium fujikuroi*	Norbikaverin	Benzoxanthentrione	Red	C_19_H_12_O_8_; 368.29	Kjær et al., 1971; Medentsev and Akimenko, 1998
58.	*Fusarium fujikuroi*	Bikaverin	Benzoxanthentrione	Red	C_20_H_14_O_8_; 382.32	Kjær et al., 1971; Wiemann et al., 2009
59.	*Fusarium fujikuroi*	β-carotene	Carotenoids	Red	C_40_H_56_; 536.87	Avalos et al., 2017
60.	*Fusarium oxysporum*	13-hydroxynorjavanicin	Naphthoquinone	Red	C_14_H_12_O_7_; 292.06	Tatum et al., 1985
61.	*Fusarium oxysporum*	1,4-naphthalenedione-3,8-dihydroxy-5,7-dimethoxy-2-(2- oxopropyl)	Naphthoquinone	Red	C15H14O7, 306.08	Tatum et al., 1985
62.	*Fusarium oxysporum*	5-O-methyljavanicin	Naphthoquinone	Red	C_16_H_16_O_6_; 304.09	Tatum et al., 1985
63.	*Fusarium oxysporum*	9-O-methylanhydrofusarubin	Naphthoquinone	Purple	C16H14O6, 302.79	Tatum et al., 1985
64.	*Fusarium oxysporum*	5-O-methylsolaniol	Naphthoquinone	Red	C_16_H_18_O_6_; 306.31	Tatum et al., 1985
65.	*Fusarium oxysporum*	8-O-methylbostrycoidin	Naphthoquinone	Red	C_16_H_13_NO^5^; 299.08	Tatum et al., 1985
66.	*Fusarium oxysporum*	8-O-methylanhydrofusarubinlactol	Naphthoquinone	Red	C_16_H_14_O_7_; 318.07	Tatum et al., 1985
67.	*Fusarium culmorum*	Aurofusarin	Naphthoquinone	Red	C_30_H_18_O_12_; 570.46	Medentsev et al., 2005
68.	*Fusarium culmorum*	Bostrycoidin	Naphthoquinone	Red	C_15_H_11_NO_5_; 285.26	Medentsev et al., 2005
69.	*Fusarium fujikuroi*	8-O-methylfusarubin	Naphthoquinone	Red	C_16_H_16_O_7_; 320.29	Studt et al., 2012
70.	*Graphis desquamescens*	Graphisquinone	Quinone	Red	C_11_H_10_O_5_; 222.19	Miyagawa et al., 1994
71.	*Graphis scripta*	Graphenone	Furandione	Yellow-orange	C_14_H_14_O_4_; 246.26	Miyagawa et al., 1994
72.	*Monascus sp*.	Monascin	Azaphilone	Yellow	C_21_H_26_O_5_; 358.43	Karrer and Helfenstein, 1932; Feng et al., 2012; Wang C. et al., 2017
73.	*Monascus rubropunctatus*	Rubropunctatin	Azaphilone	Orange	C_21_H_22_O_5_; 354.39	Haws et al., 1959
74.	*Monascus purpureus*	Monascorubramine	Azaphilone	Red	C_23_H_27_NO_4_; 381.46	Fielding et al., 1960
75.	*Monascus rubropunctatus*	Rubropunctamine	Azaphilone	Red	C_23_H_30_O_5_; 386.48	Fielding et al., 1960
76.	*Monascus purpureus*	Monascorubrin	Azaphilone	Orange	C_23_H_26_O_5_; 382.45	Fielding et al., 1960
77.	*Monascus sp*.	Ankaflavine	Azaphilone	Yellow	C_23_H_30_O_5_; 386.48	Mapari et al., 2006
78.	*Monascus purpureus*	Monapilol A	Azaphilone	Orange	C_23_H_29_O_5_; 385.20	Hsu et al., 2011
79.	*Monascus purpureus*	Monapilol B	Azaphilone	Orange	C_21_H_25_O_5_; 357.17	Hsu et al., 2011
80.	*Monascus purpureus*	Monapilol C	Azaphilone	Orange	C_26_H_33_O_6_; 441.22	Hsu et al., 2011
81.	*Monascus purpureus*	Monapilol D	Azaphilone	Orange	C_24_H_29_O_6_; 413.19	Hsu et al., 2011
82.	*Penicillium multicolor*	Pencolide	Maleimide	Yellow to orange	C_9_H_9_NO_4_; 195.174	Birkinshaw et al., 1963
83.	*Penicillium frequentans*	Questin	Hydroxyanthraquinone	Yellow to orange-brown	C_16_H_12_O_5_; 284.26	Mahmoodian and Stickings, 1964
84.	*Penicillium rubrum*	Mitorubrin	Azaphilone	Yellow	C_21_H_18_O_7_; 382.4	Büchi et al., 1965
85.	*Penicillium purpurogenum*	Purpurogenone	Quinone	Yellow-orange	C_29_H_20_O_11_; 544.10	Roberts and Thompson, 1971
86.	*Penicillium phoeniceum*	Phoenicin	Toluquinone	Yellow	C_14_H_10_O_6_; 274.23	Steiner et al., 1974
87.	*Penicillium cyclopium, Penicillium viridicatum*	Xanthomegnin	Dihydroisocoumarin	Orange	C_30_H_22_O_12_; 574.48	Stack and Mislivec, 1978; Frisvad et al., 2004,
88.	*Penicillium cyclopium, Penicillium viridicatum*	Viomellein	Quinone	Reddish-brown	C_30_H_24_O_11_; 560.50	Stack and Mislivec, 1978; Frisvad et al., 2004
89.	*Penicillium paraherquei*	Atrovenetin	Furanone	Yellow	C_19_H_18_O_6_; 342.34	Ishikawa et al., 1991
90.	*Penicillium sp. AZ*.	PP-V, 12-carboxylmonascorubramine	Azaphilone	Purple-red	C_23_H_25_NO_6_; 412.17	Ogihara et al., 2000
91.	*Penicillium sp. AZ*.	PP-R, 7-(2-hydroxyethyl)-monascorubramine	Azaphilone	Purple-red	C_25_H_31_N; 426.22	Ogihara et al., 2000
92.	*Penicillium oxalicum*	Arpink red™	Anthraquinone	Red	C_25_H_26_O_14_	Sardaryan, 2002; Dufossé et al., 2005; Caro et al., 2017
93.	*Penicillium bilaii*	Citromycetin	Chromene	Yellow	C_14_H_10_O_7_; 290.22	Capon et al., 2007
94.	*Penicillium bilaii*	Citromycin	Chromene	Yellow	C_13_H_10_O_5_; 246.21	Capon et al., 2007
95.	*Penicillium bilaii*	(–)-2,3-dihydrocitromycetin	Chromene	Yellow	C_14_H_12_O_7_; 292.24	Capon et al., 2007
96.	*Penicillium bilaii*	(–)-2,3-dihydrocitromycin	Chromene	Yellow	C_13_H_12_O_5_; 248.07	Capon et al., 2007
97.	*Penicillium purpurogenum*	N-glutarylmonascorubramine	Azaphilone	Purple-red	C_28_H_33_NO_8_; 511.23	Mapari et al., 2009
98.	*Penicillium purpurogenum*	N-glutarylrubropunctamine	Azaphilone	Purple-red	C_26_H_29_NO_8_; 483.20	Mapari et al., 2009
99.	*Penicillium marneffei*	Monascorubrin	Azaphilone	Orange	C_23_H_26_O_5_; 382.45	Woo et al., 2014
100.	*Penicillium aculeatum*	Ankaflavine	Azaphilone	Yellow	C_23_H_30_O_5_; 386.48	Krishnamurthy et al., 2018
101.	*Phycomyces blakesleeanus*	β-carotene	Carotenoids	Yellow-orange	C_40_H_56_; 536.87	Garton et al., 1950
102.	*Talaromyces funiculosus*	Ravenelin	Xanthone	Yellow	C_14_H_11_O_5_; 259.06	Padhi et al., 2019
103.	*Trichoderma aureoviride*	Chrysophanol	Hydroxyanthraquinone	Orange-red	C_15_H_10_O_4_; 254.2	De Stefano and Nicoletti, 1999
104.	*Trichoderma harzianum*	Emodin	Hydroxyanthraquinone	Yellow	C_15_H_10_O_5_; 270.2	Lin et al., 2012
105.	*Trichoderma harzianum*	Pachybasin	Hydroxyanthraquinone	Yellow	C_15_H_10_O_3_; 238.3	Lin et al., 2012
106.	*Trypethelium eluteriae*	Trypethelonamide A	Naphthoquinone	Yellow	C_23_H_29_NO_5_; 400.21	Basnet et al., 2019
107.	*Trypethelium eluteriae*	5′-hydroxytrypethelone	Naphthoquinone	Violet red	C_16_H_16_O_5_; 289.10	Basnet et al., 2019
108.	*Trypethelium eluteriae*	(–)-trypethelone	Naphthoquinone	Violet red	C_16_H_16_O_4_; 372.29	Basnet et al., 2019
109.	*Trypethelium eluteriae*	(+)-trypethelone	Naphthoquinone	Violet red	C_16_H_16_O_4_; 372.29	Basnet et al., 2019
110.	*Trypethelium eluteriae*	(+)-8-hydroxy-7-methoxytrypethelone	Naphthoquinone	Violet red	C_17_H_19_O_5_; 303.12	Basnet et al., 2019

**Figure 2:**
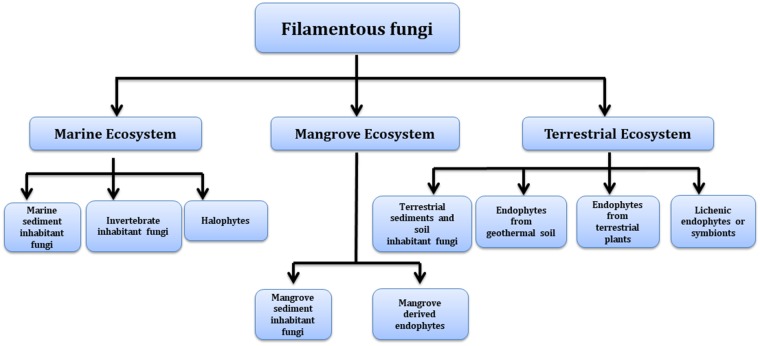
Different environments as a source of pigment producing fungi.

Many of these natural pigments are found to have a range of pharmacological activities and help fungi in various biological roles such as compounds acting as enzyme cofactors (flavins) (Rao et al., 2017); prevention against the harmful effects of photo-oxidation (carotenoids) (Gmoser et al., 2017) and the protection against environmental stress (melanins) (Dufosse et al., 2014). Though these fungal pigments have been found to be associated with diverse biological activities, the physiological role and factors regulating their production are largely unstudied (Sen et al., 2019). Recent advances in analytical and biotechnological tools employing computational and molecular means helps in deciphering the components responsible for color production, their *de novo* pathway and genome responsible for its production. Concurrently, alternative routes for mass production of these metabolites may be achieved using heterologous expression. Manipulation of culture conditions and co-culturing can also help to enhance the expression and yield of a particular pigment. Fungi capable of producing pigments can be sourced from diverse environmental conditions and lend them to be explored as a source of commercial pigments (Figure 2).

## Different Sources of Fungi Producing Pigments

### Marine Ecosystems

Fungi derived from marine environments have higher diversity and a unique scaffold of secondary metabolites which helps in their survival and their presence in extreme environmental circumstances such as absence of light, high salinity, high pressure, and low temperature. These circumstances lead to the development of extremophile microorganisms with the capacity to produce some unique molecules (Coker, 2016; Duarte et al., 2019). Fungal isolates from differential marine environment represent a major source of undiscovered pigment potential and should be target areas for commercial investigation.

#### Marine Sediment

*Penicillium bilaii* is a marine derived fungi isolated from the Huon estuary (Port Huon, Tasmania, Australia) yielding a yellow colored fungal pigment known as citromycetin and citromycin together with two dihydro analogs, (–)-2,3-dihydrocitromycetin and (–)-2,3-dihydrocitromycin (Capon et al., 2007). Similarly, *Microsporum* sp., isolated from the sample collected from Golmae Village (Ulsan City, Korea) was found to produce yellow compound flavoglaucin (Li et al., 2006). Aspergiolide B and (+)-Variecolorquinones A metabolites having red and yellow color, respectively, have been isolated from *Aspergillus glaucus* collected from marine sediment surrounding mangrove roots of Fujian Province (China) (Du et al., 2008). A water soluble red color pigments was also reported from a fungus isolated from the marine sediment sample collected from Miramar (India). The sample was identified as *Penicillium* sp NIOM-02. Release of red pigment around the colony on malt extract agar (MEA) plate indicated its hydrophilic behavior (Dhale and Vijay-Raj, 2009). In a recent study, *Talaromyces* spp., and *Trichoderma atroviride* strains obtained from marine sediment were identified as potential red pigment producers (Lebeau et al., 2017). *Talaromyces albobiverticillius* 30548, a red pigment producer strain was also isolated from sediment obtained from Reunion Island (Indian Ocean). Most of the compounds identified from this strain have been characterized as azaphilones (Venkatachalam et al., 2019). With the interest in both red and yellow hues for food colouration marine sediments are an area that could be targeted for the identification of further pigments.

#### Invertebrate Inhabitant Fungi

Sessile and non-sessile invertebrates such as corals, sponges and squirts, present in marine environment are often brilliantly colored. This bright color present in these invertebrates may be derived from photosynthetic pigments of symbiotic microorganism present in these organisms. Xu et al. (2008) has investigated fungi isolated from tropical coral reefs in order to study the extent of the bioactive molecules they contain. The team was able to isolate two novel yellow colored hexahydroanthrones named tetrahydrobostrycin and 1-deoxytetrahydrobostrycin together with red pigment bostrycin from the *Aspergillus* sp. strain of fungus isolated from coral reef off Manado, Indonesia (Xu et al., 2008). Similarly, ethyl acetate extract of the fungus *Eurotium cristatum (*ECE), isolated from the marine sponge Mycale sp., furnished a yellow colored compounds 2-(2′, 3-epoxy-1′,3′-heptadienyl)-6-hydroxy-5-(3-methyl-2-butenyl) benzaldehyde and 1,8-dihydroxy-6-methoxy-3-methyl-9,10-anthracenedione (physcion) (Almeida et al., 2010). In a recent study, Fouillaud and her team collected coral rubble and living coral from underwater volcano slopes, hard substrates, open water and sediments from different marine environments around Reunion Island in the Indian Ocean (Fouillaud et al., 2017). *Talaromyces albobiverticillius* (B and C) strains isolated from these samples were explored for pigment production and were found to generate an array of pigments unveiling specific orange/red hues under submerged fermentation in potato dextrose broth (PDB) (Venkatachalam et al., 2018). The team has isolated 42 colored compounds highlighting a range of promising hues and molecules. These isolates belong mainly to the *Aspergillus, Penicillium* and *Talaromyces* genera in the family of *Trichocomaceae* (Fouillaud et al., 2017) and as such are key species for industrial scale production. *Alternaria* is also known for its bioactive metabolites, most of which are anthraquinone derivatives which are mostly pigmented molecules having wide range of biological activities. Zheng et al., has isolated several hydroanthraquinones and anthraquinone dimers from the *Alternaria* sp. ZJ-2008003, a fungi found in soft coral of South China Sea. Some of these are bright pigmented molecules having red, pink and yellow hues (Zheng et al., 2012) which are further evidence of the broad range of colors available from invertebrate inhabiting fungus.

#### Halophytes

Microorganisms that can survive and grow well in areas of high salt (NaCl) concentration are known as halophilic extremophiles. Halophilic fungi possess great significance in biotechnological applications due to their ability to produce ample amounts of extracellular metabolites (Oren, 2010; Singha, 2012; Ali et al., 2014). Melanins are one such class of pigmented molecules and hold an important position in various cosmetic and pharmaceutical applications. The halophilic fungal strain *Trimmatostroma salinum* and *Phaeotheca triangularis* isolated from the halophiles of eastern coast of the Adriatic Sea, produce melanin pigments in solutions of saturated concentrations of sodium chloride (Kogej et al., 2004). Similarly, it was also found that a diffusible dark pigment is released on potato dextrose agar by the black yeast named *Hortaea wernecki*. This pigment holds great importance as medicinal value owing to its activity against *Salmonella typhi* and *Vibrio parahaemolyticus* (Rani et al., 2013). Further a distinctive isolate collected from a hypersaline water sample from Puerto Rico was identified as new species of *Periconia* genus and found to produce an unusual blue pigment (Cantrell et al., 2006). Halotorelant fungi have also been reported to contain some bright colored quinone compounds. Variecolorquinones A and B are two yellow colored quinone compounds that have been obtained from a halotolerant fungal strain *Aspergillus variecolor* B-17 (Wang et al., 2007). With the capacity to produces colors into the blue range the halophytes are of interest to the food industry in order to extend the color spectrum available to them.

### Mangrove Ecosystem

Mangrove ecosystems afford a remarkably diverse habitat presenting unique properties of both marine and terrestrial environment in a single ecosystem. However, the fluctuating saline and tidal habitat, extreme stress conditions and high temperature of the mangrove allows only a limited number of species that can survive in such hostile environment (Kathiresan and Bingham, 2001). Thus, the species inhabiting this intimidating environment offers an extremely affluent reserve for significant novel and biologically active compounds. However, only a small amount of mangrove fungi have been studied so far in spite of their potential for production of array of natural pigments (Das et al., 2002; Zhang et al., 2012). Some of the key findings from these reports are described in the following sections.

#### Mangrove Sediment

Chintapenta et al. (2014) has isolated ~100 mangrove fungi from the Godavari mangroves of India. Most of the strains were found to be pigment producers however special interest toward a red color directed further study on *Penicillium*, which was then interogatted for optimization of its media conditions and the effect of metals and salts on pigment yield. The main purpose of the study was to optimize the effect of various bioelements (Chintapenta et al., 2014) and it was shown that a higher concentration of salt has a negative effect on pigment yield. This is due to the fact that the presence of these electrolytes alter the pH of the medium and prevent diffusion of pigment. On the other hand, calcium, iron, zinc enhanced the pigment yield and those correlates well with typically beneficial elements that form part of plant growth.

#### Mangrove Derived Endophytes

Mangrove endophytic fungi encompasses the second largest assemblage of marine fungi and support the host plants by releasing some unprecedented metabolites which protect them from various harsh geoclimatic conditions. These endophytic fungi have been known for their various prospective applications in biotechnological and pharmaceutical field owing to the presence of structurally unique bioactive and diverse biomolecules including pigments. In an attempt to study the diversity of endophytic fungi from the tropical mangrove species*, Rhizophora mucronata*, 78 fungal isolates harboring inside the leaf tissues have been identified. Some of these isolates displayed distinctive pigmentation and are also reported to deliver a range of biological activities. These isolates produce an array of color such as green, gray/black, brown, orange, yellow, purple, and violet (Hamzah et al., 2018). Similarly, an endophytic fungus of the *Alternaria* sp., isolated from fruit of the mangrove tree *Aegiceras corniculatum* (South China Sea) is able to produce bright colored anthraquinone based compounds having hues of yellow to red. These compounds were identified as altersolanol A, alterporriols C–M, macrosporin, dactylariol, tetrahydroaltersolanol B and physcion (Huang et al., 2011). In an attempt to isolate potent radical scavenging compounds from endophytic fungi an isolate of *Eurotium rubrum* has been cultured from the inner tissue of the stem of the mangrove plant *Hibiscus tiliaceus* from Hainan Island (China). Several new pigmented components has been isolated from this fungi including questin and 3-O-(α-D-ribofuranosyl)-questin having an orange shade, asperflavin having a yellow shade, and the 2-O-methyleurotinone having a brown shade (Li et al., 2009). Similarly the *Eurotium Sp*.has also been obtained from leaves of a mangrove plant *Porteresia coarctata* (Roxb) and led to the isolation of two colored compounds identified as tetrahydroauroglaucin (yellow) and isodihydroauroglaucin (orange) (Gawas et al., 2002).

### Terrestrial Ecosystem

Terrestrial systems are a rich source of filamentous fungi and the presence of the fungi in a particular system such as sediments, soil, and decaying matter and as endophytes are mostly correlated with the production of some unique metabolites. Terrestrial microbes are also good sources of pigment producing fungi and have been relatively more explored than the aforementioned systems.

#### Terrestrial Sediments and Soil

Terrestrial sediments and soil supports the growth of filamentous fungi because of a lower level of mechanical disturbances and sheer forces that may disrupt fungal mycelia. Several studies revealed the production of pigmented components from fungi collected from soil sediments. In a study, soil sample collected from a volcanic ash from Japan yielded four bianthraquinones and two monoanthraquinones compounds having orange-red hues. These compounds were found predominantly in various soils samples collected from Japan and Nepal (Fujitake et al., 1998). An Australian terrestrial isolate of *Penicillium striatisporum* collected near Shalvey, New South Wales yielded yellow pigments. These pigments were identified as citromycetin, citromycin, dihydro analog (–)-2,3-dihydrocitromycetin (Capon et al., 2007)*. Fusarium verticillioides* isolated from soil (Chiang Mai, Thailand) was found to be a potential producer of naphthoquinone pigment (Boonyapranai et al., 2008). In a study conducted to investigate and optimize the production of pigments in submerged culture of *Penicillium purpurogenum* DPUA 1275, a strain isolated from soil samples was found to release yellow, orange, and red color (Santos-Ebinuma et al., 2013). Similarly, bioprospecting of fungi collected from an Amazon soil for the possibility of pigment production yielded five strains *Penicillium sclerotiorum* 2AV2, *Aspergillus calidoustus* 4BV13, *Penicillium sclerotiorum* 2AV6, *Penicillium citrinum* 2AV18, and *Penicillium purpurogenum* 2BV41. All of these strain were able to produce pigmented molecules however, *P. sclerotiorum* 2AV2 produced intensely colored pigments (Dos Reis Celestino et al., 2014). With the intention to evaluate the capacity of ascomycetous fungi as a promising source for the production of various components including color, Lebeau et al. (2017) has analyzed 15 ascomycetous fungal strains, out of which 11 fungal strains were of terrestrial origin. Out of all of these two terrestrial strains *Penicillium purpurogenum rubisclerotium* and *Fusarium oxysporum*, and two marine strains identified as *Talaromyces* spp., and *Trichoderma atroviride* were identified as potential red pigment producers (Lebeau et al., 2017). Recently, a cold adapted fungal strain of *Penicillium sp*. (GBPI_P155) isolated from the high altitude soil of Indian Himalayan region was reported to produce dark orange pigment. This extracted pigment was also found to be active against actinobacteria and several Gram-positive and Gram-negative bacteria (Pandey et al., 2018).

#### Endophyte Fungi From Terrestrial Plants

Endophytic fungi isolated from higher plants are a lucrative source of bioactive molecules and as such are gaining considerable attention from industries and various natural product chemists. Consequently, the numbers of scientific investigations have focussed on the isolation and identification of novel endophytes from these plants in order to obtain various bioactive molecules. These endophytic fungi are also known to produce various pigmented molecules which are mostly associated with certain specific biological activity of plants (Kaul et al., 2012). An endophytic fungus *Phyllosticta capitalensis* harbor as a foliar endophyte in a number of geographic regions tending to be hosted mostly in woody trees. To investigate the production of melanin production in this endophytic fungus, Suryanarayanan and co-workers have isolated this strains form diverse locations such as dry deciduous forests, moist deciduous forests and semi-evergreen forests. The production of melanin in the hyphae of *P. capitalensis* found to be liable for the fitness and survival of this fungus as a cosmopolitan endophyte, was shown to produce the melanin in order to help in sustaining the of fungi in stressful environments (Suryanarayanan et al., 2004). Similarly, a strain SX01, obtained from the twigs of *Ginkgo biloba*, was found to be a strong producer of red pigment which can be used as a producer of natural food additive (Qiu et al., 2010). In a similar study, *Aegle marmelos* which is a medicinal tree acclaimed for curing a range of disorders was investigated for the isolation of endophytic fungi. This experiment led to isolation of 169 endophytic fungal strains and importantly 67 among those were found to be pigmented (Mani et al., 2015).

#### Endophytes From Plants Inhabiting Geothermal Soil

Geothermal ecosystems are an exclusive combination of scarce microclimatic and edaphic environments which provides distinctive habitats enabling the survival of unique vegetation. The environmental stresses of these areas includes elevated soil temperatures, elevated air temperatures, humidity, excess alkalinity, acidity and the presence of higher concentrations of some metals such as aluminum which eliminates most vascular plant species from surviving in these conditions. Species which can survive such circumstances are mostly unique to these specific regions or have some unique features which enable them to survive in such adverse conditions (Smale et al., 2018). Redman et al. (2002) proved that the presence of mutualistic fungi particularly endophytic fungi in *Dichanthelium lanuginosum* assist in the survival of plant in geothermal soil and is the basis for the thermo-tolerance to the plant. The author also inferred that thermal protection in plants could be due to the production of the fungal pigment melanin which aids in the dissipation of heat along the hyphae (Redman et al., 2002).

#### Lichens and Endolichenic Fungi

Among the broad range of microbial resources, lichens are the ones which are gaining importance and have become the focus of significant pharmaceutical companies due to the presence of unique metabolites (Stocker-Wörgötter et al., 2013; Calcott et al., 2018). Lichens are microbial assemblages having a close symbiotic relationship between the fungal partner known as mycobiont and a photosynthetic algal partner known as photobionts (Culberson and Culberson, 1970; DePriest, 2004). In this association the fungal partner protects the algal partner from adverse geoclimatic influences. One way this protection is provided is the production of pigmented molecules by the fungal partner which are known to be effective in shielding algal partner from ultraviolet radiation (Nybakken et al., 2004; Nguyen et al., 2013). However, the slow growth rate of lichen in nature is a major hurdle to obtain these pigments for commercial exploitation. Considering this, recent reports have described obtaining pigments from cultured mycobionts opening new pathways for commercialization of these compounds from lichens (Stocker-Worgotter, 2008; Calcott et al., 2018). Miyagawa et al. (1994), isolated two novel pigments, identified as graphisquinone and graphenone from the cultured mycobionts of the lichens *Graphis desquamescens* and *Graphis scripta* and these are red quinone and a yellow-ornage furandione, respectively (Miyagawa et al., 1994).

It has also been observed that some of the isolated lichen mycobionts produce new metabolites under laboratory driven stressed conditions such as osmotic stress, which are otherwise not produced by the natural lichen thallus (Miyagawa et al., 1993). Moriyasu et al.'s team (2001) found a bright yellow pigment yielded by the spore-derived mycobiont culture from a lichen of the *Haematomma* sp. (Moriyasu et al., 2001). Importantly this pigment could not be found in natural sample with the aid of traditional chemical analysis. It was also observed that some mycobionts release fluorescent pigments which were otherwise not present in lichen thallus growing naturally. A cultured mycobiont from the lichen *Amygdalaria panaeola* released three new fluorescent pigments namely panaefluorolines A–C (Kinoshita et al., 2003) extending the color range and utility of these types of sources to industrial applications. A wide range of hues and shades have been reported from the mycobiont of lichen *Trypethelium eluteriae* which includes a yellow pigment, trypethelonamide A and a novel dark violet red pigment 5′-hydroxytrypethelone along with three known dark violet-red pigments (–)-trypethelone, (+)-trypethelone and (+)-8-hydroxy-7-methoxytrypethelone (Basnet et al., 2019). Isolation of endolichenic fungi from the lichen thallus has also attracted attention for their potential to produce a range of bioactive molecules including pigments. A recent study on the isolation of bioactive molecules from the endolichenic fungus *Talaromyces funiculosus* yielded three compounds including ravenelin which is a yellow colored homogeneous powder that also possess good antimicrobial activity thus making it advantageous to the pharmaceutical and food sectors (Padhi et al., 2019).

## Current Challenges in Harnessing the Potential of Fungi

Although, there are a plethora of fungal resources which can serve as a source of potential pigments it is important to note that there are several challenges faced by industry which curtail the process of commercialization. Sustainability and progression of the fungal based pigment industry is mainly reliant upon three important factors (a) absence of mycotoxin in fungal pigments (b) pigment yield (c) pigment stability and purity (Lebeau et al., 2017).

Food grade pigments need approval through regulatory agencies and the most important precondition in their consideration is toxicological safety of the product (Food Administration Drug, 2015; Sigurdson et al., 2017). Most of the fungi producing pigments are known to synthesize some toxic metabolites known as mycotoxins along with the pigments. The production of these mycotoxins irrefutably restricts the application of these pigments either directly in food or as an additive owing to its own safety issue (Dufossé et al., 2005). A well-known example is the production of toxin citrinin along with Monascus pigments which posed a challenge over its safe use and thus was prohibited in the European Union and the United States (Carvalho et al., 2005). Another major challenge in fungal pigments is the pigment yield. The range of chemical entities and the range of properties limit its profitable yield with targeted isolation methodologies (Chadni et al., 2017). Pigment yield in a culture can be enhanced by increasing the biomass growth or by enhancing the accumulation of intracellular pigments (Das et al., 2007; Dufosse et al., 2014). Medium optimization which includes monitoring of operating conditions such as media composition, temperature, agitation, aeration and pH are critical parameters to regulate in order to reach optimum pigment production (Mondal et al., 2015). The cost effectiveness of the selected media is also an important parameter to consider and at scale is particularly important for industrial processing. Another important challenge is the stability of natural pigments against environmental factors such as pH, light, moisture, temperature and food matrices (Ogbonna, 2016) where a shorter shelf life due to instability of molecule at varied conditions may limit its application as commercial pigments. However, such stability issues can be addressed with the help of novel approaches such as microencapsulation (Ersus and Yurdagel, 2007; Özkan and Bilek, 2014) and nanoformulation (Mehrad et al., 2018). These formulations can help to improve the physical properties and stability which have been routinely used for other purposes in the food industry including milk processing.

Technology that use shell materials as the basis for the microencapsulation of pigments include freeze-drying, spray-drying, emulsion and coacervation. Spray-drying is the most widely used technique in food industry for pigment stabilization and increases the shelf life of the product significantly. Several studies have discussed the food applications of microencapsulated natural colorants (Azeredo et al., 2007; Ersus and Yurdagel, 2007) which have been broadly accepted by the industry. However, there is another technique called asnanoencapsulation, the application of this technique is limited in industries till date. Nanoencapsulation can be employed to enhance the stability and solubility issues associated with natural pigments. The considerably small size of nanoemulsions and nanocapsules make them a useful vehicle for the distribution of non-polar pigments in aqueous solutions of those from fungal sources. Matsuo and his co-workers in a recent study prepared two types of nanoparticles encapsulating *Monascus* pigments employing a hydroxypropyl cellulose (HPC) and poly (lactic-co-glycolic) acid (PLGA) copolymer. These formulations were found to enhance the photostability of the *Monascus* based pigment and drew the attention of researchers in this field. It is important to note however that the PLGA based nanoparticle were effective toward improving the stability upon photobleaching of the pigments as compared to the HPC nanoparticles (Matsuo et al., 2018). Application of these approaches to enhance the solubility and stability of natural pigments as per the requirement based on food commodity could be a promising area of research for further advancement.

## Biotechnological Advances in Pigment Production

### Metabolomics

A number of technologies to overcome issues associated within the pigment industry are already in place and many are in the process of implementation. The recent advent of biotechnological based approaches have been established toward intelligent screening methods for the selection of appropriate strains and exploit the traditionally overlooked potential of pigment production by various fungal strains. A step stage in the use of biotechnological approaches has been observed within the industry in the past decade with a focus on the execution of different ways for intelligent screening (excluding toxic producing strains) which has been shown to increase the yield of pigment production (Mapari et al., 2005). Screening of possible pigment-producing fungal strains with the help of metabolomic tools helps in clustering strains on the basis of their characteristic metabolites including functional groups associated with color and also allows for some control over the selection of the strains with known toxic metabolites (Hajjaj et al., 1999; Archer, 2000). Approaches involving the latest data handling methods and chemoinformatics tools for the identification of metabolites help to perform a systematic study of these molecules in target species. These studies not only assist in dereplication of already known molecules but also help in targeting novel pigments with a chromophore similar to already established pigments (Elyashberg et al., 2002). An example of using automated techniques for the targeted screening of molecules of interest has been performed with the aid of computerized screening which has led to the novel production of monascus like pigments. An approach using X-hitting algorithm was shown to be useful when applied to the UV-vis spectra of metabolites (cross hits). The tool has been used as a quick way to screen ascomycetous filamentous fungi belonging to *Penicillium* subgenus *Biverticillium* which is not reported to produce citrinin or any other mycotoxin (Mapari et al., 2008).

### Metabolic Engineering

**Figure 3:**
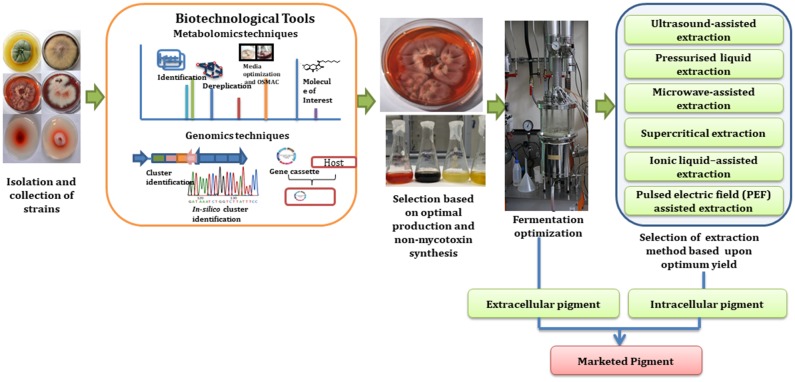
Schematic roadmap for a pigment journey from lab scale to industrial scale (Subimage—fermenter picture has been adapted from “https://commons.wikimedia.org/wiki/File:Institut_de_chimie_des_substances_naturelles_de_Gif-sur-Yvette_en_2011_109.jpg” under the “Labeled for reuse” right.

Besides the identification of metabolites with these mass-metabolomics techniques, genome knowledge also helps in identification of desired secondary metabolites (Arora, 2003). Recent advances in molecular biology and metabolic engineering has helped in streamlining the process of pigment industrialization as described in Figure 3. For instance, tools involved in molecular biology helps in sequencing of fungal genomes and thus assist in identification of genes involved in production of pigmented metabolite (Sankari et al., 2018). The use of genome mining strategies for the discovery of new pigmented molecules is one of the most constructive techniques as it not only allows studying the complete metabolic capacity of fungal strain but also allows studying the gene clusters that are not expressed in standard laboratory culture conditions (Nielsen and Nielsen, 2017). These gene clusters can then be engineered in controlled ways for the overproduction of a desired pigment or expressed in a heterologous host for the large scale production of selected pigments (Pfeifer and Khosla, 2001; Jiang et al., 2010). Cloning the genes and encoding for a selected pigments biosynthesis into microbial vectors such as *Bacillus subtilis, E. coli, Corynebacterium glutamicum, Pseudomonas putida*, and *Pichia pastoris* has been considered as a more reliable and cost effective approach for industrial production process. Production of carotenoids mainly β-carotene and torularhodin has been enhanced in *Rhodotorula mucilaginosa* KC8 by using metabolic engineering and a mutagenesis approach (Wang Q. et al., 2017). Further, novel betalain derivative production has been shown to be induced in *Saccharomyces cerevisiae* as a heterologous microbial host by using glucose as a substrate and by using different amines in the culture (Grewal et al., 2018). This is particularly important as it leads to control of the biological process in an industrial setting and could be the key to realizing the potential of this field.

In a recent study conducted by Chen and co-workers, production of monascin and ankaflavin pigments have been studied by knockout of the *mrPigF* gene and elucidation of the MonAzPs biosynthesis in *Monascus ruber* M7. The findings in this study provided a roadmap toward the selective and controlled biosynthesis of the desired MonAzPs constituents (Chen et al., 2017). Similarly, Balakrishnan et al. (2013) studied the azaphilone pigment biosynthetic gene cluster using the T-DNA random mutagenesis in *Monascus purp*ureus. It was found that transcription of transcriptional regulator gene (mppR1) and the polyketide synthase gene (MpPKS5) was significantly repressed in the W13 albino mutant. Additionally, targeted inactivation of MpPKS5 gave rise to an albino mutant, validating the role of mppR1 and MpPKS5 toward azaphilone pigment biosynthesis. Importantly the MpPKS5 gene cluster includes SAT/KS/AT/PT/ACP/MT/R domains, which is also preserved in other azaphilone polyketide synthases. Likewise, six azaphilone compounds, azanigerones A–F have also been linked with aza gene cluster in *Aspergillus niger* ATCC 1015. This was confirmed with the help of transcriptional analysis and deletion of a key polyketide synthase (PKS) gene (Zabala et al., 2012) where whole genome expression analysis together with existing knowledge of polyketide synthase (PKS) genes helped in identification of three novel clusters of co-expressed genes in *F. verticillioides*. With the help of functional analysis of PKS genes linked to these clusters, a violet pigment in sexual fruiting bodies (perithecia) and the mycotoxins fusarin C and fusaric acid were identified (Brown et al., 2012). In an another study, attempt was made to identify the gene responsible for the production of 1,8-dihydroxynaphthalene-melanin pigment (DHN-melanin) in *Ascochyta rabiei*. Degenerate PCR primers were used to obtain an *ArPKS1* which is encoding for a polypeptide with high similarity to polyketide synthase (PKS) involved in biosynthesis of DHN-melanin in other ascomycetous fungi through site-directed mutagenesis of *ArPKS1* in *A. rabiei* generated melanin-deficient pycnidial mutants confirming the linkage of *ArPKS1* with melanin production (Akamatsu et al., 2010). Two strains of *Streptomyces galilaeus* ATCC 31133 and ATCC 31671 known to produce anthracyclines namely aclacinomycin A and 2-hydroxyaklavinone, anthraquinones, aloesaponarin II after transformation with DNA from *Streptomyces coelicolor* containing four genetic loci, actI, actIll, actlV, and actVII (Bartel et al., 1990). Identification of gene cluster and elucidation of the promoters regulator using various molecular biology tools such as sequencing of fungal genomes would help in studying the molecular aspects of pigment generation. These advances will also assist in understanding the complexity associated with the biosynthetic pathways of pigment metabolites which can be exploited for industrial application.

### Culture Optimization

The culture environment presents a range of critical parameters than can be studied in order to control the composition and yield of fungal secondary metabolites. One Strain Many Compounds (OSMAC) is a well-known model which has highlighted the concept of changes in the fungal secondary metabolism with respect to growth conditions (Romano et al., 2018). Thus, media optimization is also a prerequisite parameter to be considered for maximizing the yield of the fermentative product. This strategy involves the abiotic modifications to the culture environment by modifying the culture medium composition such as altering the source of carbon and nitrogen and controlling the operating parameters like the redox status, temperature, light intensity, wavelength, culture configuration (aeration, agitation, static, liquid, or solid) and pH. In an attempt to identify the effect of media composition on pigment yield the effects of glutamic acid on the production of monacolin K pigment and expression of the monacolin K biosynthetic gene cluster has been studied. The presence of glutamic acid medium in spite of the original medium increased the monacolin K production from 48.4 to 215.4 mg l^−1^ which is equivalent to 3.5 times. Upregulation of the expression of mokB-mokI; on day 8 in the presence of glutamic acid was the driver behind this upsurge (Zhang et al., 2017). Beyond the substrate and culture conditions, bioreactor design also plays a significant role in optimizing the process of pigment production and this is an area that the process industry will be able to add their expertise for the realization of efficient controlled natural pigment manufacture (Zhong et al., 1992; Spier et al., 2011).

## Processing and Extraction of Natural Pigments

Filamentous fungi are a great source of wide range of metabolites such as polyphenols, polyketides, carotenoids and terpenoids (Rao et al., 2017). Selection of extraction techniques is one of the crucial step for the efficient recovery of these metabolites and mostly reliant upon the nature of metabolites of interest and localization of these metabolites in the fungal culture (Chadni et al., 2017). Some of the pigments are extracellular and released in the fermentation broth which makes their extraction downstream less trivial than the intracellular pigments that requires specialized extraction techniques for their removal from biomass (Morales-Oyervides et al., 2017). Introduction of cheap, efficient and safe extraction methodology for the recovery of natural pigments is one of the major challenges to be overcome in order to enable production at a large scale. A series of conventional techniques which includes organic solvent extraction (soxhlet, homogenization, and shaking), hydrodistillation, centrifugation extraction, and steam distillation have been worked upon in order to extract pigments from various fungi although the upscaling of these processes is a non-trivial exercise. Nevertheless, the quest of biotech industries for unearthing the safer extraction technologies over the last two decades fuelled a tremendous amount of research toward the development of newer greener extraction and separation methods. Once these major issues have been addressed by the industry, focus can be placed on the challenge of keeping the process cheap, efficient and fast.

### Extraction of Extracellular Pigments

For the purpose of easy and feasible downstream processing, extracellular and water soluble pigments obtained from the submerged culture are preferred (Velmurugan et al., 2010). Water soluble pigments do not require any organic solvents for their extraction thus are considered safe and also can be used directly in different food commodities without further modification or engaging any carriers/stabilizers. Conversely, intracellular and water insoluble compounds requires conventional extraction processes with organic solvents, which are not only complicated (due to safety and environmental impact) and time-consuming processes but also bring the need for more costly and rigorous regulatory controls. Therefore, removing these tedious extraction processes would help in alleviating the use of large amount of solvents, which not only reduce the production time but also eliminates the cost of an extraction process and assists in making the pigment production more economical and safer and environmentally friendly (Hu et al., 2012). Extraction of extracellular pigments through submerged culture is mainly controlled by two parameters fermentation optimization and fermentation processing which we shall focus on in the next two sections.

#### Fermentation Optimization

**Figure 4:**
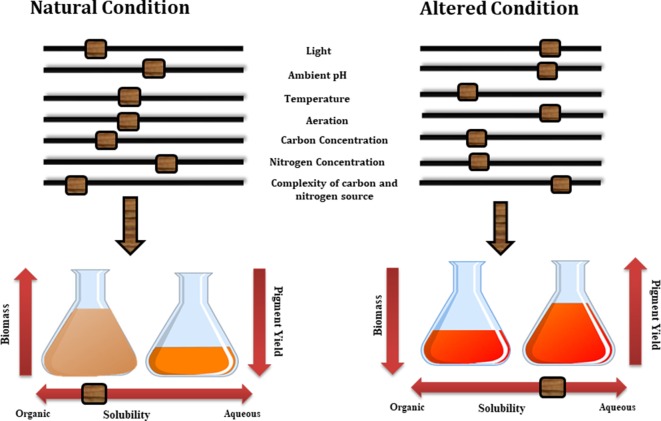
Various factors effecting the growth of biomass and pigment yield.

Although the cellular mechanism behind the secretion of these metabolites are still not known a number of studies have highlighted the effect of various growth conditions, such as medium composition and process parameters on the nature and yield of these metabolites. Thus by altering various growth conditions in submerged culture such as pH, aeration, light exposure, concentration of carbon and nitrogen in the medium and their ratio, production of extracellular and water soluble pigments can be altered (Figure 4) (Hajjaj et al., 1998). For example, changes in the pH of culture medium of *Monascus* sp. alter the concentration of extracellular pigments in particular (Mukherjee and Singh, 2011). Similarly, Lebeau et al. (2017) emphasized the fact that medium composition plays a very important role for the selection of extracellular pigments. In their study the effect of simple and complex forms of carbon sources on the mycelium biomass and extracellular pigment production has been discussed and it was concluded that simple form of carbon such as defined minimal dextrose broth (DMD) favors the production of higher biomass but low pigment production. Additionally the complex form of carbon and nitrogen sources present in potato dextrose broth (PDB) along with essential cofactors including magnesium, calcium, iron, phosphor, zinc, manganese and copper (all present in PDB broth) encourages the production of extracellular pigments over biomass production.

A successful industrial bioprocess development relies on the optimization step which includes optimization of biomass yield and its bioactive production with stimulating metabolic precision. On industrial scale, two major submerged fermentation techniques are mostly applied in order to discover metabolites of interest by amplifying their production from a laboratory to large scale. First is fed-batch approach (Caro et al., 2017) which primarily works like batch fermentation until the exhaustion of one or more substrates which are then renewed by addition of fresh medium through a targeted feeding regime. A fixed volume or variable volume of substrate or medium is then used accordingly (Li et al., 2000; Amanullah et al., 2010). Krairak et al. (2000) and Chen et al. (2015) have used the fed-batch fermentation technique and optimized the condition required for the maximizing yellow pigment yield from different *Monascus* sp. Their studies provided an appropriate fermentation strategy to produce high proportion of yellow pigments in high cell density culture (Krairak et al., 2000; Chen et al., 2015). On the other hand the continuous mode approach works on the delaying of the exponential phase by feeding the microbial cells with fresh nutrients and the cells are routinely collected from the bioreactor at the defined rate and reaction time point (Vogel et al., 2012). Both of these techniques are also popular among pigment industry with the implication that together with suitable optimization process would result in production of large amount of extracellular pigments which can be easily collected or processed without solvent extraction.

#### Fermentation Processing

Aqueous two-phase system (ATPS) is one of the most popular methods used for the extraction of pigmented compounds from the fermented broth (Iqbal et al., 2016). This approach uses liquid-liquid fractionation and is based upon the use of green ionic liquids for extraction of pigmented molecules. Separation of different hydrophilic solutes into two immiscible aqueous phases is mainly based upon their differential selectivity toward different polymer–polymer, polymer–salt, or salt–salt and solute combinations formed in these two phases. Aqueous two-phase systems offers several advantages for downstream control of biomolecules (1) both the phases are composed of water compared to organic solvents in conventional extraction thus provides favorable environment to molecules and supports the stability of their structure and bioactivity, (2) an environmental friendly process, (3) are economically favorable (McQueen and Lai, 2019).

Several researchers have implemented ATPS extraction approaches for the isolation of pigments from submerged culture broths. Ventura and co-workers implemented ATPS based on quaternary ammonium and imidazolium system for the recovery of red pigment from the fermented broth of *Penicillium purpurogenum* DPUA 1275. The purpose of the study was to separate red pigment molecule from the remaining colorants and contaminant proteins present in the fermented broth. They have concluded that the optimal extraction system based upon tetraethylammonium bromide ionic liquid assisted in the high partition coefficients of the red pigment (K_red_ = 24.4 ± 2.3) and protein removal (EE_Total_ =60.7 ± 2.8 %) from the fermentation broth (Ventura et al., 2013).

### Extraction of Intracellular Pigments

For the extraction of hydrophobic compounds and intracellular compounds, green extraction methods are more preferable as they are either free from organic solvents or need fewer amounts of solvents and thus are considered more safe and environment friendly. Some of these extraction techniques also work on low temperature which also helps in the extraction of thermolabile pigments without their degradation. These extraction techniques include ultrasound-assisted extraction (UAE) (Vilkhu et al., 2008; Cheung et al., 2013), pulsed electric field (PEF) assisted extraction (Goettel et al., 2013; Fincan, 2017), pressurized liquid extraction (Lebeau et al., 2017, 2019), microwave-assisted extraction (MAE) (Vázquez et al., 2014), ionic liquid–assisted extraction (Ventura et al., 2013; Grosso et al., 2015; Lebeau et al., 2016) and supercritical CO_2_ extraction (Cocks et al., 1995; Chaudhari, 2013). Most of these techniques have been tested for the extraction of bioactive compounds from numerous natural resources. Only few reports are available for the extraction of carotenoids pigments using green extraction methods from raw plant materials, microalgae and seaweeds (Poojary et al., 2016). The implication of all these novel extraction techniques in extraction of fungal based pigments with or without involvement of GRAS (generally regarded as safe) solvents could be an interesting avenue to be explored further by research teams and industry.

#### Ultrasound-Assisted Extraction (UAE)

Ultrasound-assisted extraction (UAE) has been well-acknowledged as an efficient and environmentally safe extraction methods in number of phyto-pharmaceutical industries (Chemat et al., 2017). Owing to the thermolabile nature of most of the natural metabolites, possibility of their degradation during thermal extraction is very high. In contrast, ultrasound-assisted extraction resulted in increased extraction efficiency at lower temperature. This method of extraction is mainly based upon the employment of high-intensity ultrasound pressure waves to accelerate the extraction of a solid material in a liquid solvent. These waves works by generating localized pressure which ultimately cause the tissue to rupture and assist in release of intracellular substances into the solvent. It is mainly working on acoustic energy which is not absorbed by molecule but is transmitted through the medium. Ultrasound waves are transmitted through medium by means of pressure waves induced vibrational motion of the molecules (Tiwari, 2015).

The major advantage of using ultrasound-assisted extraction is its faster kinetics which further contributes toward enhanced extraction yield. The other benefit of using UAE is that its apparatus is simple and cheap and is quite easy to use compared to other novel extraction techniques. Further, UAE can be done with a smaller amount of solvent which helps in extracting a wide variety of bioactive compounds including those that are water soluble (Vilkhu et al., 2008).

#### Pressurized Liquid Extraction (PLE)

Pressurized liquid extraction (PLE) also known as accelerated solvent extraction (ASE), is a very recent extraction technique and has emerged as an advanced technique to conventional solvent extraction methods such as reflux, Soxhlet extraction, percolation or maceration in terms of solvent consumption, extraction time, extraction yields and reproducibility (Mustafa and Turner, 2011). It is an automated technique used for the exhaustive extraction from solid matrices with the help of elevated pressure and temperature and diminishing solvent consumption. Both the parameters work together for the complete removal of metabolites from matrix and the high pressure assists in the penetration of solvents in the sample while with the help of higher temperature the solubility and diffusion rate of the metabolites is enhanced by breaking the interactions between matrix and analytes (Richter et al., 1996). Application of the elevated conditions in PLE helps in the reduction of total extraction time and consumption of extraction solvents. Recently, PLE has been shown to be the most popular technique for extraction of bioactive molecules from plants and fungi owing to its advantage to preserve the closest possible compositions of the molecule (Camel, 2001). In a recent study performed by Lebeau et al. (2017), extraction of intracellular pigments from the fungal biomass was performed using six-stage pressurized liquid extraction (PLE). Extraction of red pigments in optimum yield from *Talaromyces* species proved PLE as a faster and greener extraction technique as compared to conventional extraction techniques (Lebeau et al., 2017)

#### Microwave-Assisted Extraction (MAE)

Microwave (MW) radiation falls between frequencies ranging from 300 MHz (radio radiation) to 300 GHz in the electromagnetic spectrum. Microwave-assisted extraction (MAE) employs microwave radiation as the source of energy to heat the sample and solvent mixture based of the dipole moments (Li et al., 2012; Xiong et al., 2016). Microwave-assisted extraction is one of the advanced extraction technique known for its efficient extraction efficiency with minimum solvent consumption and lower extraction time. This technique is mostly used for the extraction of high-value bioactive compounds present in various biological matrices and helps in producing high quality extracts (Pare et al., 1991). Factors which are considered to demonstrate an important role in the extraction and separation efficacy and selectivity of MAE are the substrate material, solvents, solid–liquid ratio, temperature, pressure, and particle size (Chupin et al., 2015).

The relevance and potential of water as the only solvent or in solvent free microwave assisted processes for the extraction of bioactive metabolites has been critically discussed by a number of reviews (Filly et al., 2016; Seoane et al., 2017). Water being the most safe, nontoxic, non-flammable, non-corrosive, and environmentally benign falls under the category of green solvent and in combination with microwave assisted water extraction helps in the extraction of wide range of metabolites from fungal and plant matrices (Flórez et al., 2015). Pasquet et al. (2011) identified MAE as the best extraction technique for the isolation of pigments from microalgal sources owing to its reproducibility rapidity, uniform heating, and high extraction yields (Pasquet et al., 2011). Similarly, some other studies have also reported potential of MAE in the extraction of pigments from plant sources and what has been learnt by these experiment can be exploited in this field (Kiss et al., 2000; Liazid et al., 2011).

#### Supercritical Extraction (SFE)

A surge in the application of supercritical extraction (with CO_2_) as a greener extraction technique in natural product chemistry was observed during the last decade (Da Silva et al., 2016). Supercritical extraction is an innovative extraction technique based upon the employment of liquefied carbon dioxide gas as the supercritical fluid for the extraction of bioactive molecules from sold matrices (Khaw et al., 2017). This technique is working on the principle of augmented solvating power of specific gases above their critical limit and thus the gas behave like a supercritical fluid having properties of liquid together with gas as extracting fluid. Combining the transport properties of a gaseous phase with density like a liquid phase provides the supercritical fluid, a cutting edge feature as a novel extracting medium. Dissolving power of these supercritical fluids is determined by the pressure and temperature employed during the extraction process which can be adjusted by manipulating these parameters (Zabot et al., 2012). One of the major advantages of using SFE for the extraction of natural metabolites is the extraction of thermolabile compounds as extraction can be performed at low temperatures. It has also been acknowledged as a green sustainable technique for the selective isolation of molecules.

However, supercritical CO_2_ is mainly used for the extraction of non-polar compounds due to its hydrophobic nature but its polarity can be tailored in combination with different co-solvent such as ethanol for the extraction of relatively polar compounds as xanthophylls and ethylene for extraction of carotenoids. Several examples of employment of SFE for carotenoids extraction from several substrates from laboratory to the commercial scale have been reported (Nobre et al., 2006; Kitada et al., 2009). Several parameters such as temperature, time, pressure, flowrate, choice of co-solvent can considerably modify the extraction yield and efficiency along with selectivity for compounds of interest. Accordingly, selection of these parameters must be judiciously taken into account for an efficient and selective recovery of target analytes.

#### Ionic Liquid–Assisted Extraction

Ionic liquids (ILs) have emerged as tailor-made solvents for the extensive extraction and purification of natural-derived bioactive compounds. It has been recognized as tuneable designer solvent owing to its diverse array of salt combination fabricated for particular range of compounds and helps to overcome the drawback of limited selectivity associated with the usage of organic solvent (Passos et al., 2014). Moreover, implementation of ILs helps to make the process more economical and also helps to diminish the environmental footprint.

These ILs in combination with water or organic solvents can be implemented directly in extraction of bioactive molecules from biomass known as simple ILs assisted SLE (Solid liquid extraction). However owing to its ionic behaviors, these ILs can interact with electromagnetic fields and therefore can be used in combination with MAE known as IL-based microwave-assisted extraction or UAE known as IL-based ultrasonic-assisted extraction (Ventura et al., 2017).

A very recent study has discussed the application of protic ionic liquids (PILs) as cell lytic agents to extract and improve the extractive yield of intracellular carotenoids from yeast *Rhodotorula glutinis* biomass (Mussagy et al., 2019).

#### Pulsed Electric Field (PEF) Assisted Extraction

Pulsed electric field (PEF) processing is a non-thermal extraction technique working on the principle of electroporation or electro-permeabilization. It is a process of exposing the sample matrix to short impulses of high intensity electric field which eventually result in cell membrane disintegration and increased its permeability. This effect can be of reversible or irreversible nature depending on process parameters which includes amplitude, intensity, number, duration and frequency of the external electric waves. Most of the secondary metabolites are positioned inside the plant cells and increased permeability of the cell membrane helps in the rapid diffusion of the solvent inside the cell and removal of these metabolites in external environment. Unipolar or bipolar pulses with square-wave shaped or exponential frequencies are mostly used in this treatment. Pulsed electric field assisted extraction can be used for the selective extraction of metabolites by manipulating the intensity of treatment which is mainly controlled by pulse duration, electric field strength, treatment time or energy input. Numbers of studies have discussed the potential of using PEF in pigments extraction from algal cells and different matrices (Grimi et al., 2014; Luengo et al., 2015). Parniakov et al. (2015) has also discussed the effect of the combination of PEF and solvent extractions containing biphasic mixture of organic solvents at different pH for the recovery of hydrophobic carotenoids and other pigments in an efficient manner. Result of their study highlighted the effect of two step extraction as high levels of extracted non-degraded proteins was recovered at the first step during PEF extraction and substantial enhancement of pigments yield at the second step. Also, this two stage PEF-assisted procedure allowed also effective extraction using lower amount of organic solvents (Parniakov et al., 2015).

## Conclusion

The major aim for the pigment industry, especially for food grade pigments is to look for a sustainable and potential source of pigments which is relatively safe for human health and the environment. The modern inclination in society for “natural” ingredients and consumer concern toward the deleterious effects of synthetic pigments on health and environment rekindled the interest toward the use of natural colorants. Progressive growth involving various biotechnological tools for the supply of nutritive, attractive healthy and high sensorial quality products has been observed in last decades which has made this process more economical and suitable for mass applications. Nature may be excellent source of safe colors, however, key limitations such as raw material availability and variation in pigment profile associated with colors obtained from plant source, navigate color industry toward the potential of colors obtained from microbial sources particularly fungal resources.

Keeping the advantages afforded by fungal diversity in mind, fungi are considered as cell factories for pigment production, where researchers can play with functionality. Fungal species obtained from various sources are known to produce and yield wide array of pigments which are usually associated with multifaceted biological activities together with extraordinary range of colors. Although the number of traditional technologies for the production of pigments such as monascin (from a fungus) are already well-advanced a lot of research on new alternatives, exploring novel means and sources for the biotechnological production of these pigments in profitable yield are in progression. Thus, further research is necessary to find optimize pigment properties such as yield and composition by optimized growth parameter, using metabolic engineering tools, introduction of low cost organic substrates for value addition, presence of different elicitors for pigment production, stabilizing methods for improving pigment application and suitable greener and environmentally safer extraction methods for the extraction at large scale.

## Author Contributions

RK contributed toward drafting and editing of the article. MG and XC contributed toward the critical revisions and final approval of the article.

## Conflict of Interest

The authors declare that the research was conducted in the absence of any commercial or financial relationships that could be construed as a potential conflict of interest.
